# A Comprehensive Hepatitis B Surface Antigen-Positive Patient-Centered Screening and Linkage to Care Strategies Targeting Microelimination of Hepatitis C Virus Infection in Chongqing, China

**DOI:** 10.1155/2022/9644576

**Published:** 2022-12-27

**Authors:** Dachuan Cai, Dazhi Zhang, Peng Hu, Hong Ren

**Affiliations:** ^1^Department of Infectious Diseases, The Second Affiliated Hospital, Chongqing Medical University, Chongqing, China; ^2^Institute for Viral Hepatitis, Chongqing Medical University, Chongqing, China; ^3^Key Laboratory of Molecular Biology for Infectious Diseases, Ministry of Education, Chongqing, China

## Abstract

**Background and Aims:**

The likelihood of coinfection increases in regions where HBV is endemic because of the similar transmission route. China is another endemic nation, with 5.9% of the population being HBsAg-positive. This study aimed to evaluate the prevalence of HCV antibody positivity in HBsAg-positive subjects, HCV RNA positivity in anti-HCV positive subjects, and HBV/HCV coinfection with the hope of exploring hepatitis C microelimination using currently available therapies.

**Method:**

12,500 HBsAg-positive serum samples were collected. All samples were screened for anti-HCV. Furthermore, positive samples were screened for HCV RNA. All patients with positive HCV RNA were followed up for suspicious transmission routes of HCV and linkage to care.

**Results:**

44 out of 10,560 (0.4%) patients with positive HBsAg had detectable anti-HCV. There were 32 males and 12 females, with a statistical difference. 17 out of 44 were HCV RNA positive. Among them, 15 out of 38 patients were HCV RNA positive; 8 patients had started anti-HCV treatment with the DAA regimen, while the other 7 patients had not. After patient education, one patient had begun treatment and reached SVR12, while three patients still refused anti-HCV treatment.

**Conclusion:**

The HCV/HBV coinfection prevalence was found to be lower in this study. Even though HBV and HCV share a somewhat similar transmission route, HBsAg-positive subjects may not be at high risk for HCV infection. The process of hepatitis C's microelimination could be accelerated by increasing patient awareness and education. This trail is registered with NCT03794791.

## 1. Introduction

Viral hepatitis C is a kind of infectious disease and a global endemic caused by the hepatitis C virus (HCV), to which the majority of the population is vulnerable. Blood is one of the main routes of pathogen transmission. After contracting an HCV infection, there is a significant risk of developing chronic hepatitis C, with estimates ranging from 55%–85%. Although it is possible that some people would not exhibit any evident signs, the general population has a 5% to 15% risk of cirrhosis if the infection has lasted for more than 20 years [1].

Recent research estimated that over 71 million people around the world are chronically infected with HCV, with approximately 10 million of these subjects residing in China [2, 3]. Notably, HBV and HCV are blood-borne viruses with similar transmission routes. Some high-risk individuals have a chance of developing HBV and HCV coinfection at some point during their lifetimes. Studies have revealed that being coinfected with hepatitis B and C at the same time (HBV/HCV coinfection) can accelerate the progression of liver disease [4–6]. However, the use of direct-acting antiviral agents (DAAs) has made it possible to treat and even cure hepatitis C.

The goal of the microelimination strategy for hepatitis C is to eradicate the disease in order to reduce the burden it places on individuals, communities, and institutions. This will be accomplished through a variety of strategies, some of which include incorporating HCV drugs into the health insurance system, increasing screening for high-risk and ordinary people in each region and in special places (such as hospitals and addiction centers), expanding health care propaganda about HCV, and increasing the rate of HCV diagnosis and linkage to care.

Taking all of these things into account, this study evaluated serum anti-HCV antibodies in HBsAg-positive patients and interviewed those who tested positive by telephone. Following that, all HCV-infected patients in this subset were additionally tested for HCV RNA. Patients who tested positive for HCV RNA were contacted by telephone to discuss their prognosis and treatment options. This was done in an effort to encourage them to undergo anti-HCV therapy.

## 2. Materials and Methods

A total of 12,500 outpatients and inpatients with positive hepatitis B surface antigen who visited the Second Affiliated Hospital of Chongqing Medical University, Chongqing, China, between June 1, 2018, and November 24, 2020, were enrolled. A blood sample of about 2.0 milliliters (ml) was collected from each patient. Repeat visits were excluded based on the patients' medical records. This research was approved by the ethical committee of the second affiliated hospital of Chongqing Medical University. (201837).

**Figure 1:**
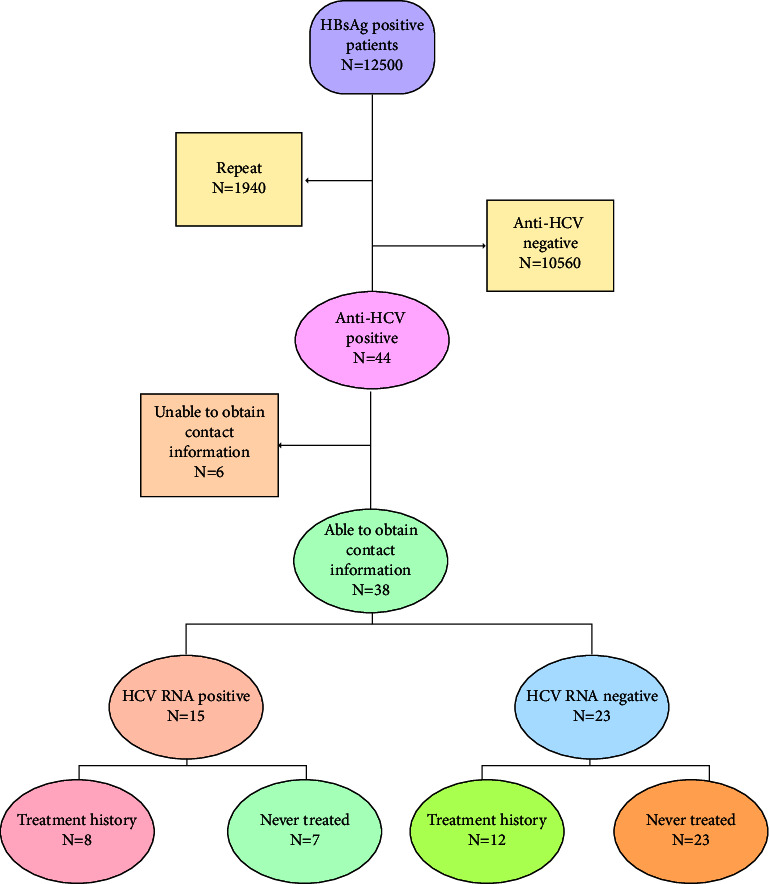
Flowchart.

All the tests followed the methods of Cai et al. [7]. First of all, the detection of HBsAg and anti-HCV antibodies followed the instructions in the user's manual provided by the manufacturers. ELISA method was used to detect serum HBsAg (Abott GmbH & Co, KG, Germany) and serum anti-HCV antibody (Shanghai Kehua Bio-Engineering Co, Ltd, Shanghai, China) in all patients. Secondly, RT-PCR was used to detect the HCV RNA loads in patients with positive anti-HCV antibodies (Light Cycler480, ROCHE, Swiss; HCV RNA nucleic acid detection kit (detection limit of 100 IU/ml), and Shanghai Kehua Bio-Engineering Co, Ltd, Shanghai, China). Patients with positive anti-HCV antibodies in Hospital Information System (HIS) were interviewed by telephone to determine their HCV cognition level, HCV treatment history, suspicious routes of HCV infection, HCV treatment willingness, and the reasons for refusing HCV antiviral treatment. HCV-related information, development of DAAs, and insurance policies were conveyed to those HCV-RNA-positive patients in order to encourage anti-HCV therapy during the telephone interview, as shown in Figure 1.

SPSS26.0 was used for statistical analysis, and the Chi-square test was used for rate comparison, while the independent sample *T*-test was used for continuous variables, and *P* < 0.05 was considered statistically significant.

## 3. Results

**Figure 2:**
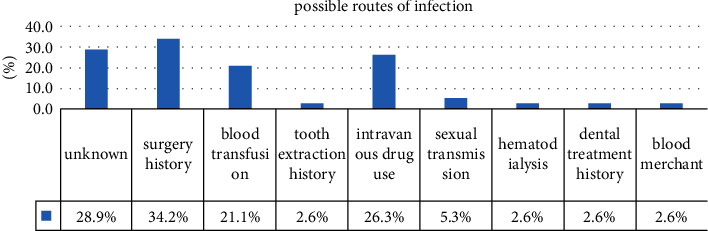
Possible routes of infection.

Based on the patients' medical records, 1,940 cases of repeat visits were excluded (Table 1). Successful patients totaled 10,560 in the end, with 6,118 males and 4,442 females. Out of 10,560 HBsAg-positive patients, 44 (0.4%) had anti-HCV antibody positivity, 17 (0.16%) had HCV RNA positivity, and 38.6% (17/44) were positive in anti-HCV antibody positive patients. However, only 15 of them (15/17) were followed up by telephone interviews or a database search. Unfortunately, only 21 patients' information about genotype was retrieved from the HIS database, including five genotypes 1, one genotype 2, six genotypes 3, and five genotypes 6. Results from the amplification for genotyping were negative in four more cases. Positive cases with anti-HCV antibodies typically exhibit the following general characteristics: the average age of 44 patients was 46.98 ± 8.77 years old, with 32(72.7%) male and 12 (27.3%) female, and the difference was statistically significant (*P* = 0.003). The average age of males was 46.34 ± 7.58 and females was 48.67 ± 11.59, and the differences were statistically significant (*P* = 0.032). 38 of the 44 cases who were contacted or whose data were available had suspected routes of getting HCV infection. These include: 13/38(34.2%) surgery history, 10/38 (26.3%) intravenous drug use, 8/38 (13.9%) blood transfusion use, 1/38(2.6%) tooth extraction history, 1/38 (2.6%) paid blood donation, 1/38 (2.6%) hemodialysis, 2/38(5.3%) sexual transmission, 1/38(2.6%) dental treatment history, and 11/38(28.9%) others (Figure 2).

23 of the 38 participants, whose treatment information was collected or with whom contact was made were negative for HCV RNA, whereas 15 were positive. 12/23 HCV RNA-negative patients had a history of anti-HCV treatment, while 11/23 HCV RNA-negative patients had no history of antiviral treatment and had spontaneous virus clearance. 15 out of 38 participants, whose information could be obtained or contacted, tested positive for HCV RNA. Eight of these patients had started HCV treatment, whereas seven had no prior HCV treatment history. One of the seven patients with no history of HCV treatment succumbed to HCC, while four failed to grasp the importance of hepatitis C treatment. After HCV-related education, one patient had already begun HCV treatment with SOF/VEL + RBV and completed treatment, whose HCV RNA at 12 weeks was negative. However, three patients still declined treatment owing to financial hardships, terminal-stage lung cancer, or lack of care. The other two patients did not begin HCV therapy because they did not know they were afflicted with HCV. However, after getting information about HCV and expressing a desire to begin treatment, DAA was planned for them at an appropriate time.

Twenty (52.6%) of the 38 patients had a history of HCV treatment, while 18 had no history of HCV treatment. Among the patients with a history of treatment, nine were treated with Indian generic drugs, 2 with sofosbuvir + darunavir ± ribavirin, 3 with sofosbuvir/vipatavir ± ribavirin, 2 with Elbasvir/Gravir, 1 with interferon + ribavirin, 2 with sofosbuvir + ribavirin, and 1 with Ledipasvir/Sofosbuvir.

One of these 38 patients tested positive for HBsAg and anti-HBc. Three were positive for HBsAg, HBeAg, or anti-HBc. 34 were positive for HBsAg, HBeAb, or anti-HBc. Three out of 38 HBV DNA test samples were positive. For the three individuals with HBV DNA positivity and negative HCV RNA, no HCV treatment was necessary. 8/20 patients with a history of anti-HCV therapy had HBV treatment prior to initiating anti-HCV medication, while 3/20 patients combined HBV treatment with anti-HCV therapy. 2 out of 20 patients initiated HBV therapy at the end of HCV therapy, with abnormal liver function and confirmed hepatocellular cancer (HCC). 7 out of 20 did not initiate HBV treatment at all.

There were four patients who developed HCC. They were all positive for HBsAg, HBeAb, and anti-HBc positive, but negative for HBV DNA. One-fourth of the population consisted of females, while three-fourths consisted of males. One-fourth of HCV RNA-positive patients have died in the absence of anti-HBV and anti-HCV treatment. One-fourth had uremia and negative HCV RNA without a history of anti-HBV and anti-HCV treatment, and they were also deceased. Two-fourths were HCV RNA negative and had consumed alcohol over 80 g per day for more than 20 years, one of them had anti-HCV therapy, and both of them started anti-HBV therapy after HCC developed.

## 4. Discussion

The treatment of chronic hepatitis C infections has been revolutionized by the recent advent of highly efficient direct-acting antivirals with cure rates above 95%, making the physical eradication of viral hepatitis C possible. Nevertheless, a substantial proportion of individuals with HCV infections might stay asymptomatic for decades, placing them at risk for a slow progression to severe liver disease and death if they do not obtain timely testing and treatment. In light of the efficacy of DAAs, the strategy for eradicating HCV has shifted from treatment to the identification of subjects with chronic HCV infection. Africa and the Western Pacific region have the highest prevalence of hepatitis B infection, between 7.5% and 5.9%, according to the World Health Organization. China, which is located in eastern Asia along the Western Pacific region and has a population of more than 1.4 billion, has the highest chronic HBV infection population in the world, with approximately 82.6 million individuals [8]. Therefore, detecting HBV infection using serological means (including HBsAg) is the standard practice in China. Since HCV is also a type of blood-borne virus just like HBV, it makes sense to test for HCV infection in subjects who already have a chronic HBV infection. However, there is no conclusive data on the proportion of subjects with HBV and HCV coinfection.

This study showed that the anti-HCV antibody positive rate was 0.4% and the HCV RNA positive rate was only 0.16% in this special population of 10,560 HBsAg-positive patients in our hospital, which was not significantly different from the anti-HCV antibody positive rate of 0.43% in the 1–59 years old Chinese population in 2006 [9], but the anti-HCV antibody positive rate was significantly lower than that in previous studies at home and abroad.

Ruiling et al. [10] found that 9.3% of 103 (10/103) HBsAg-positive patients in China's Shaanxi Province, blood donors were also HCV-positive. The anti-HCV positive rate in chronic hepatitis B (CHB) patients in China was 11.39∼14.47 percent, according to a previous epidemiological investigation [11, 12]. In an Iranian investigation involving 139 HBsAg-positive patients, the rate of anti-HCV antibody positivity was 12.3% [29]. According to data from Italy [13–15], Egypt [16], the United States [17, 18], and Turkey [19], the incidence of HCV infection among patients with chronic hepatitis B ranges from 0.55% to 9.5%. The HBV/HCV coinfection rate in our study is significantly lower than that in the rest of China and other nations, which may be due to the following factors: First of all, this study is based on a hospital-based patient population rather than a community-based one. Secondly, even though HBV and HCV share a somewhat similar transmission route, HBV is transmitted vertically in Chinese patients while HCV is transmitted horizontally. Abuse of intravenous drugs is strongly outlawed in China due to the fact that this is the primary mode through which HCV is spread horizontally. Therefore, it is possible that the prevalence of anti-HCV antibodies among HBsAg-positive patients in Europe and the United States is higher than it is in China. Thirdly, fewer subjects were included in the above domestic and foreign studies than in our study, which may also have an impact on the data on coinfection with HBV and HCV. In fact, the approach that is now being taken in this research endeavor does not intend to discover the majority of HCV cases, which is something that needs to be done by screening high-risk groups. The present method prioritizes “microelimination,” which refers to the process of locating each and every case using all means at one's disposal.

Among the 38 patients who tested positive for anti-HCV antibodies, the results showed that 20 of them (52.6% of the total) had previously received anti-HCV treatment, while the remaining 18 patients had no history of anti-HCV treatment. Furthermore, 7 of the patients tested positive for HCV RNA, while 11 of the patients tested negative for HCV RNA. This suggests that the 11 patients who tested negative for HCV RNA had a history of HCV infection but may have undergone spontaneous HCV clearance. In adults, the rate of chronicity following HCV infection has been estimated to range anywhere between 50 and 80 percent in previous studies. According to the findings of our research, 27 out of 38 (71.1%) individuals had chronic hepatitis C, which was in line with findings from previous studies. One of the seven patients who tested positive for HCV RNA but had no prior history of therapy passed away, whereas the other four out of seven did not have any clinical manifestations.

The identification of patients alone is not sufficient for the microelimination of HCV infection. This was understood through patient telephoned interviews when patients and their families knew nothing about HCV infection and its clinical consequences, or whether there was therapy available. This was found to be the reason why patients refused anti-HCV therapy. In this study, among the 4 untreated patients who did not realize the importance of hepatitis C treatment, 1 patient had accepted DAA therapy for hepatitis C, had completed the treatment, and achieved SVR12 after the patient education (in the form of telephone interview), while 3 patients still refused treatment due to financial constraints, terminal stages of lung cancer, or the belief that there were no symptoms, therefore, there were no diseases. This revealed that the lack of HCV infection symptoms and patients' understanding of the disease's chronic nature are the two main factors impeding the eradication of the virus. Therefore, it is important to raise public awareness of the disease and publicize the benefits and costs of the current treatment for hepatitis C. In this study, it was also found that two other patients did not start HCV treatment because they did not know they had contracted HCV. After being informed of the virus nature, HCV, and the disease, hepatitis C, during the telephone interview, they had decided to take DAA for hepatitis C treatment in the near future. It is obvious that now that there are so many patients with HBV infection in China and they should undergo a routine blood test, it is possible to have the test of HCV infection included in the routine HBV test to improve the detection rate and treatment rate of hepatitis C in China.

The majority of the 20 patients with a history of HCV therapy opted for less expensive generic medications rather than the original formulations. From January 2020 on, sofosbuvir/velpatasvir and elbasvir/grazoprevir have been included in the national medical insurance in China, and the individual burden for the cost of anti-HCV treatment has been approximately one-seventh of the previous level, allowing more patients with HCV infection to access standard HCV care. There is no doubt that patient education is the most effective method for providing them with information.

In this study, 15 out of 38 individuals tested positive for HCV RNA. Among these 15 individuals, eleven did not have a history of HBV or HCV treatment despite having negative HBV DNA. Compared to a single infection, HBV/HCV coinfection may result in a higher incidence of liver cirrhosis, liver decompensation, and liver cancer [20]. It has been demonstrated that successful anti-HCV therapy and SVR are advantageous for reducing the severity of hepatic fibrosis and the incidence of HCC [21–27]. Therefore, patients with HCV and HBV coinfection should start antiviral therapy as early as possible. In this study, there were four out of thirty patients developed HCC. All of them were HBsAg, HBeAb, anti-HBc positive, and HBV DNA negative, aged between 39 and 54, without HBV treatment. Only one of them had HCV treatment before, while others neither had HBV treatment nor HCV treatment.

There are also some limitations to this study. First of all, this study explored the status of HBV/HCV coinfection mainly in hospital visited patients. Those who did not visit a doctor were not included. Secondly, some medical history data of patients with positive anti-HCV antibodies and HCV RNA was missing due to a wrong phone number or the uncooperative attitude of those patients, and thus we failed to educate them about HCV through the telephone.

In conclusion, China has the most HCV RNA-positive cases of any country in the world, with about 200,000 cases reported each year. Hepatitis C can now be cured. To microeliminate hepatitis C, it is important to raise awareness, lower the cost of treatment, and manage and track patients' treatment plans. This meets the WHO's 2030 viral hepatitis elimination objective [28]. In addition, more real-world studies of HBV/HCV coinfection are needed to explore HCV elimination in the HBsAg-positive population.

## Figures and Tables

**Table 1:** General demographic characteristics of cases with HBsAg and anti-HCV antibody positive (*N* = 44).

Variables		No. of cases %	HCV-RNA positive
Sex (*n* = 44)	Male	32 (72.7%)	14 (82.4%)
	Female	12 (27.3%)	3 (17.6%)

Age (*n* = 44)	21∼30	1 (2.3%)	0 (0%)
	31∼40	7 (15.9%)	3 (17.6%)
	41∼50	23 (52.3%)	9 (52.9%)
	51∼60	10 (22.7%)	2 (11.8%)
	61∼70	3 (6.8%)	3 (17.6%)

HBV status (*n* = 44)	HBsAg+, HBeAg+, anti-HBc+	4 (9.1%)	1 (25%)
	HBsAg+, HBeAb+, anti-HBc+	39 (88.6%)	16 (41.0%)
	HBsAg+, and anti-HBc+	1 (2.3%)	0

Duration of HBV therapy in patients receiving anti-HCV therapy (*n* = 20)	Before anti-HCV therapy	8	
	Simultaneously with anti-HCV therapy	3	
	After HCV therapy	2	
	Not start HBV therapy	7	

Liver cirrhosis status (*n* = 40)	Decompensated cirrhosis	10	
	Compensated cirrhosis	7	
	No liver cirrhosis	23	

Genotype (*n* = 21)	GT1	5	
	GT2	1	
	GT3	6	
	GT6	5	
	No amplification	4	

Probable infection routes (*n* = 38)	Intravenous drug use	10 (26.3%)	6 (60.0%)
	Blood-borne	1 (2.6%)	1 (100.0%)
	Surgery history	13 (34.2%)	5 (38.5)
	Tooth extraction history	1 (2.6%)	0
	Sexual transmission	2 (5.3%)	1 (50.0%)
	Transfusion	8 (21.1%)	3
	Dental treatment history	1 (2.6%)	1 (100.0%)
	Hemodialysis	1 (2.8%)	0
	Unknown	11 (28.9%)	2 (18.2%)

Treatment history (*n* = 20)	Others	9 (45.0%)	
	SOF + DCV ± RBV	2 (10.0%)	
	SOF/VEL ± RBV	3 (15.0%)	
	Elbasvir/grazoprevir	2 (10.0%)	
	IFN + RBV	1 (5.0%)	
	SOF + RBV	2 (10.0%)	
	Ledipasvir/sofosbuvir	1 (5.0%)	

SOF = sofosbuvir; DCV = daclatasvir; VEL = velpatasvir; IFN = interferon; RBV = ribavirin.

## Data Availability

The data that support the findings of this study are available from the corresponding author upon reasonable request.
